# Assessment of specific versus combined purpose knowledge based models in prostate radiotherapy

**DOI:** 10.1002/acm2.12483

**Published:** 2018-10-19

**Authors:** Elizabeth Bossart, Melissa Duffy, Garrett Simpson, Matthew Abramowitz, Alan Pollack, Nesrin Dogan

**Affiliations:** ^1^ Department of Radiation Oncology University of Miami Miller School of Medicine Miami Florida; ^2^ Department of Biomedical Engineering University of Miami Coral Gables Florida

**Keywords:** knowledge based planning, model library, prostate cancer, radiation therapy, treatment planning

## Abstract

Knowledge‐based planning (KBP) can be used to improve plan quality, planning speed, and reduce the inter‐patient plan variability. KPB may also identify and reduce systematic variations in VMAT plans, something very important in multi‐institutional clinical trials. Training of a KBP library is a complex and difficult process, and models must be validated prior to their clinical use. The purpose of this work is to assess the quality of the treatment plans generated using a specific versus combined purpose model KBP library for prostate cancer. Seven KBP model libraries were created from a set of patients treated on various Institutional Review Board (IRB) approved protocols. All KBP libraries were validated using an independent set of twenty patients (half treated Pr: Prostate alone half treated PLN: prostate plus pelvic lymph nodes). Two models were tested on the Pr patients only, four tested on PLN patients only, and one tested on all patients. All plans were normalized such that at least 95% of the prostate planning target volume received 100% of the planned dose. The plans based on different model libraries were compared to each other and the expert clinical plan. For Pr plans there were almost no statistically significant differences (*P *<* *0.008) between the plans types except conformity index (CI) with library plans better than the expert. For PLN plans, all model libraries in generally showed femur doses and CI better than the expert plans (*P *<* *0.003). This study demonstrated that no large differences were observed between specific versus combined KBP model libraries in dosimetry of prostate cancer patients. This would allow for a fewer specific plans to be needed to create a model library. Further studies are needed to evaluate benefits of combined purpose model libraries for planning of complex sites such as head and neck cancer.

## 1 INTRODUCTION

Both intensity modulated radiotherapy (IMRT) and volumetric modulated arc therapy (VMAT) are advanced technologies that have been commonly used for treatment of prostate cancer.^1^, ^2^ However, there may be large variations in the quality of the treatment plans due to differences in both experience and skill of the treatment planners. Such differences may limit the desired organs‐at‐risk (OAR) sparing and the target coverage that can be achieved. Recent investigations have demonstrated that knowledge based planning (KBP), which utilizes a library of previously treated patient plans, has enormous potential for improving the quality and consistency of treatment plans.^1^, ^2^, ^3^, ^4^, ^5^, ^6^, ^7^, ^8^

KBP library models are a way to objectively incorporate years of prior solid expert planning experience into the planning process. KBP allows planners of all experience levels to predict the best possible planning goals as well as to create treatment plans that draw on the lessons from successful prior plans.^3^, ^4^ If created properly, such libraries have the power and potential to shorten the time it takes to produce high quality treatment plans as well as to making it possible for planners at all experience levels to generate such plans. A tremendous amount of work goes in to creating these models. Many things must be considered in choosing plans for the models including the quality of the contours, the variations in size for the organs at risk (OARs) and target volume(s), the types of treatment plan (including field setup, energy used, and treatment technique), the overall quality of the plan, the dose goals reached by the plan, and finally the prescription level or levels used in creation of the plan.^9^

Initial evaluation of KBP has demonstrated that KBP is able to generate clinically acceptable plans for the treatment of prostate cancer.^1^, ^10^ However, a question arose during the creation of models for prostate planning as to the appropriateness of including plans of more than one type in the model. For instance, does creating a model containing plans that treat prostate alone alongside plans that treat prostate plus pelvic lymph nodes outperform a model that includes only one of those two plan types? A search of the literature showed no answer as to whether it was either preferable or necessary to use plans of a single type in the model in order to get the best results. The drawback of only being able to use a single plan type is clear: one would need to have a large number of single purpose plans in order to create a viable model. Being able to include plans of more than one type means that an initial model can be created with fewer plans overall. This study seeks to answer the question of whether the use of a combination of different plan types in the creation of KBP model libraries produces plans of the same quality when compared to a KBP model that consists only of plans of a specific type.

## 2 MATERIALS AND METHODS

### 2.A Patient selection

Ninety‐seven Volumetric Modulated Arc Therapy (VMAT) plans were selected from a database of patients who were enrolled in various IRB‐approved protocols for treatment of prostate alone (Pr) or prostate plus pelvic lymph nodes (PLN). For all prostate patients on these protocols, RapidArc (ver. 13.7, Varian Medical Systems, Palo Alto, CA, USA) plans had been created with 10 MV or 23 MV photons utilizing 2–4 full arcs. The goal for both arms of the protocol was to cover at least 95% of the PTV volume with 100% of the prescription dose while limiting the volume of PTV, where possible, to doses no greater than 110%. For the trials, the Clinical Target Volume (CTV) was defined as the Gross Tumor Volume (GTV) plus between 0.25 and 1 cm of the proximal seminal vesicles (SVs). The PTV included a 5 mm expansion in all directions except posterior where it was expanded by 3 mm. All patients had 3–4 gold fiducial seeds or tracking markers placed in the prostate under ultrasound guidance prior to the treatment planning CT. The planning CT is then typically co‐registered to a planning MRI for contouring guidance. Organs at Risk (OAR) delineated include anus plus rectum (defined as AnoRectum), bladder, penile bulb, and sigmoid colon or other bowel lying within 2 cm of the PTV. A very strict bowel/bladder preparation was followed prior to both imaging for treatment planning and each treatment fraction that is described elsewhere in the literature.^11^ The various clinical trials for prostate alone had prescriptions to the prostate PTV of 36.25 Gy in 5 fractions, 70.2 Gy in 26 fractions and 80 Gy in 40 fractions. The PLN plans had a prescription of 80 Gy in 40 fractions to the prostate PTV while treating the lymph nodes to 56 Gy in the same number of fractions.

All the clinical plans were generated by three very experienced planners based on the PTV and OAR constraints per protocol with goals and constraints listed in Table 1. Variations in the quality of the plans included in the models were due mostly to the individual patient variations, planner skill level, and time allotted for planning even though the optimization objectives for PTVs and OARs during RapidArc planning are strictly enforced.

**Table 1:** Dosimetric constraints for planning

Structure	Planning goals
Bladder	No more than 25% receives 81.25% PIV
	No more than 50% receives 50% PIV
Anus/Rectum	No more than 17% receives 81.25% PIV
	No more than 35% receives 50% PIV
L/R Femur	Maximum point dose of 62.5% PIV
Bowel	Less than 150 cc to receive 50% PIV
PTV (prostate)	At least 95% of the volume to receive PIV
PTV (LNs)	At least 95% of the volume to receive 70% PIV

PIV, Prostate PTV Prescription Isodose Volume.

### 2.B KBP model configuration and training

A KBP optimization tool, called RapidPlan (ver. 13.7, Varian Medical Systems, Palo Alto, CA, USA) was employed in this study. The RapidPlan tool is used to create KBP models. RapidPlan consists of a statistical model generated from the geometries of PTVs and OARs as well as the dose distributions created for previously treated patients to predict a range of achievable dose‐volume histograms (DVHs) for OARs of new patients. The detailed description of the components of the Eclipse knowledge based optimization engine (RapidPlan), including model building and training and automated model based DVH estimation tool have been discussed in several publications^4^, ^8^, ^12^ and will not be repeated here.

Seven KBP model libraries were generated using instructions provided by the RapidPlan user guide. These consisted of patients as follows: (a) n = 66 patients treated to the prostate alone (Pr); (b) n = 31, subset of (c) patients treated to the prostate plus lymph nodes (PLN); (c) a combined library with n = 97 patients which includes all patients from libraries (a) and (b) (PPLN); (d) a combined library with n = 66 to match the size of (a), where *P* = 35 patients and PLN = 31 patient (P35PLN31); and (e–g) three combined libraries, all subsets of (c) with n = 31 to match the size of (b) where (e) where *P* = 20 patients and PLN = 11 patients (P20PLN11), (f) where *P* = 16 patients and PLN = 15 patients (P16PLN15) and (g) where *P* = 11 patients and PLN = 20 patients (P11PLN20). Once the models were configured, the outlier analysis was done using the model analytics tool provided by Varian Medical Systems. All plans in the model libraries were calculated using the Acuros XB (ver. 13.7) dose calculation algorithm.

### 2.C Model validation

Twenty patients (10 Pr alone, and 10 PLN) that were not included in any of the KBP library‐training sets were used for model validation. Models (a) and (d) were validated using the ten Pr alone patients, models (b), (e), (f) and (g) were validated using ten patients treated to the prostate plus lymph nodes, and model (c) was validated using all 20 patients. Validation means that a new treatment plan was generated for each of these patients utilizing a single run of the RapidPlan optimizer with minimal planner intervention utilizing each appropriate KBP library. All treatment plans were examined for quality with both physicians and physicists comparing qualitatively and quantitatively the plans.

### 2.D Plan evaluation

The plans generated using different KBP libraries were compared to each other and the clinical plans using the PTV dose coverage and OAR sparing based on the dose‐volume parameters listed in Table 1. Specifically, the data points taken on each plan were the minimum, maximum and mean doses to the PTV80 and, where appropriate, PTV56 treatment volumes; the maximum and mean doses to the bladder, AnoRectum, and both femoral heads; and the percent volume of the bladder and AnoRectum receiving 80, 65 and 40 Gy. The conformity index (CI) and homogeneity index (HI) were also evaluated. CI was defined by the Radiation Oncology Therapy Group (RTOG) as

$$CI_{\mathrm{RTOG}}=PIV/TV$$

where PIV is the prescription isodose volume and TV is the tumor volume.^13^ The HI used here is defined as

$$HI=D_{2\%}-D_{98\%}/D_{p}$$

where D_2%_ is the dose to 2% of the PTV, D_98%_ is the dose to 98% of the PTV and D_p_ is the prescription dose for the PTV.^14^ In general, the closer CI is to the value of one, the better (more conformal) the plan, and the closer HI is to the value zero, the better (more homogeneous) the plan.

Wilcoxon signed‐rank test was used to do pairwise analysis for the statistically significant differences between plans generated using different KBP libraries and clinical plans. As there was a comparison of 4 plan subtypes for prostate alone plans (Expert, Pr, PPLN, P35PLN31; 6 comparison tests), to account for Type‐I statistical errors (or false positives), a *P*‐value of <0.0083 (two‐tailed) was considered statistically significant. As there was a comparison of 6 plan subtypes for prostate plus lymph node plans (Expert, PLN, PPLN, P20PLN11, P16PLN15, P11PLN20; 15 comparison tests), to account for Type‐I statistical errors, a *P*‐value of <0.0033 (two‐tailed) was considered statistically significant.

## 3 RESULTS

All plans generated by the original KBP model libraries were considered clinically acceptable for treatment within the guidelines set out in the treatment protocols, meaning they met or exceeded the goals in Table 1. Tables 2 and 3 list the plan comparisons for Pr alone and PLN, respectively, which showed statistical significance (*P* < 0.008 for prostate alone and *P* < 0.003 for prostate plus lymph nodes).

**Table 2:** Plan parameters with significant differences in pairwise Wilcoxon rank‐sum testing of Pr alone plans

Dosimetric data point	Plan comparison (*P* value)	Result
Mean AnoRectum	Expert vs Pr (0.0046)	Expert plan has lower average Anorectum dose
CI_RTOG_	Expert vs Pr (0.0028)	Pr plan more conformal
	Expert vs P16PLN15 (0.0022)	P16PLN15 plan more conformal
	Expert vs PPLN (0.0008)	PPLN plan more conformal

**Table 3:** Plan parameters with significant differences in pairwise Wilcoxon rank‐sum testing of PLN plans

Dosimetric data point	Plan comparison (*P* value)	Result
Min PTV80	Expert vs P20PLN11 (0.0013)	P20PLN11 Min dose lower than expert
Max L Femur	Expert vs PLN (0.0002)	In all cases, Expert Max L Femur dose higher
	Expert vs PPLN (0.0002)	
	Expert vs P20PLN11 (0.0002)	
	Expert vs P16PLN15 (0.0002)	
	Expert vs P11PLN20 (0.0002)	
Max R Femur	Expert vs PLN (0.0002)	In all cases, Expert Max R Femur dose higher
	Expert vs PPLN (0.0004)	
	Expert vs P20PLN11 (0.0002)	
	Expert vs P16PLN15 (0.0002)	
	Expert vs P11PLN20 (0.0002)	
Mean PTV80	Expert vs PLN (0.0017)	In all cases, Expert Mean PTV80 dose higher
	Expert vs PPLN (0.0003)	
	Expert vs P20PLN11 (0.0002)	
	Expert vs P16PLN15 (0.0002)	
	Expert vs P11PLN20 (0.0002)	
Max L Femur	Expert vs PPLN (0.0007)	In all cases, Expert Mean L Femur dose higher
	Expert vs P20PLN11 (0.0010)	
	Expert vs P16PLN15 (0.0002)	
	Expert vs P11PLN20 (0.0017)	
Max R Femur	Expert vs PPLN (0.0017)	In all cases, Expert Mean R Femur dose higher
	Expert vs P20PLN11 (0.0013)	
	Expert vs P11PLN20 (0.0028)	
CI_RTOG_ (PTV80)	Expert vs PPLN (0.0003)	In all cases, Expert had CI farther from “ideal” (KBP plans more conformal)
	Expert vs P20PLN11 (0.0002)	
	Expert vs P16PLN15 (0.0028)	
HI (PTV80)	Expert vs PLN (0.0008)	In all cases, Expert had HI farther from “ideal” (KBP plans more homogeneous)
	Expert vs PPLN (0.0003)	
	Expert vs P20PLN11 (0.0002)	
	Expert vs P16PLN15 (0.0002)	
	Expert vs P11PLN20 (0.0002)	

Note that on the list in Table 2 there are no comparisons of statistical significance between two different model libraries for prostate alone plans, only between the expert planners and a KBP model library. The only significant OAR datapoint for prostate alone plans was that the expert planner had a lower mean AnoRectum dose than the Pr alone KBP model. Otherwise, all significant differences were for CI with the KBP model plans being generally more conformal, and thus more ideal, than the expert plans. The expert plans had a CI_RTOG_ of 1.06 on average, and each KBP model had plans averaging a CI_RTOG_ of 1.01.

Note as well that on the list on Table 3 there are also no comparisons of statistical significance between two different model libraries for the prostate plus lymph node plans, only between the expert planners and KBP model library. For each of the model libraries used for prostate plus lymph node cases, the maximum femur doses (both left and right) and the PTV80 mean doses were lower for the KBP models than the expert planners. In all comparisons of homogeneity index, the KBP model library plans were more homogeneous than the expert plans. For most library types, the mean femur doses were lower for the KBP library, and the CI was closer to ideal for the KBP model library plans more often than not. In only one case, for library P20PLN11, the minimum dose to the PTV80 was greater by about 750 cGy, on average, for these plans than for the expert plans.

There were no statistically significant differences between in plan quality when comparing the large sized model (n = 97) and the smaller sized models (n = 66 or n = 31); as well as no difference between the models with different ratios of cases types for the various PPLN models.

**Figure 1:**
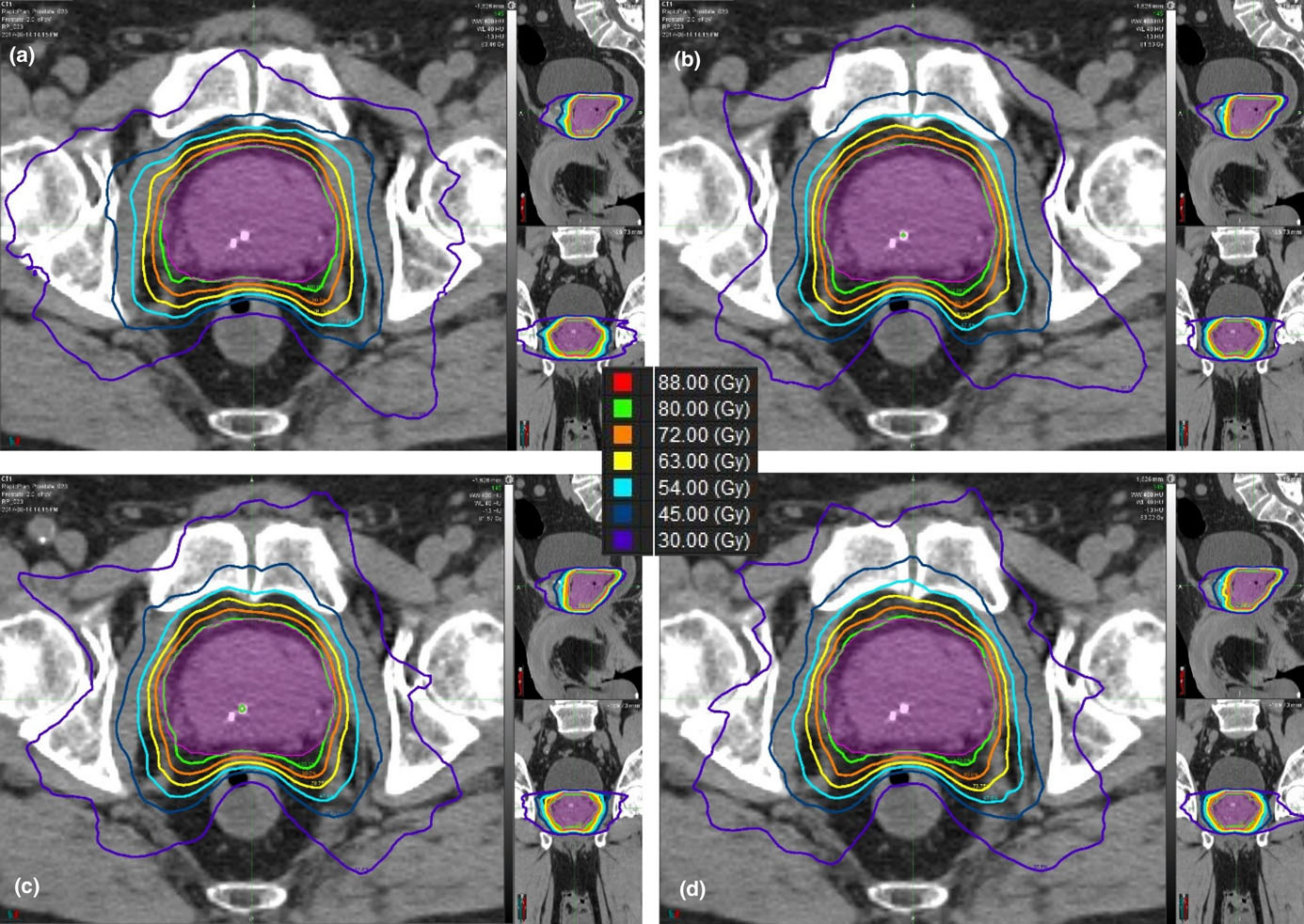
Example patient plans for prostate alone utilizing (a) Expert planner, (b) Prostate alone KBP Model (Pr Alone), (c) full prostate plus pelvic lymph node KBP Model (PPLN), and (d) the KBP Model with 35 prostate cases and 31 prostate plus lymph node cases (P35PLN31).

**Figure 2:**
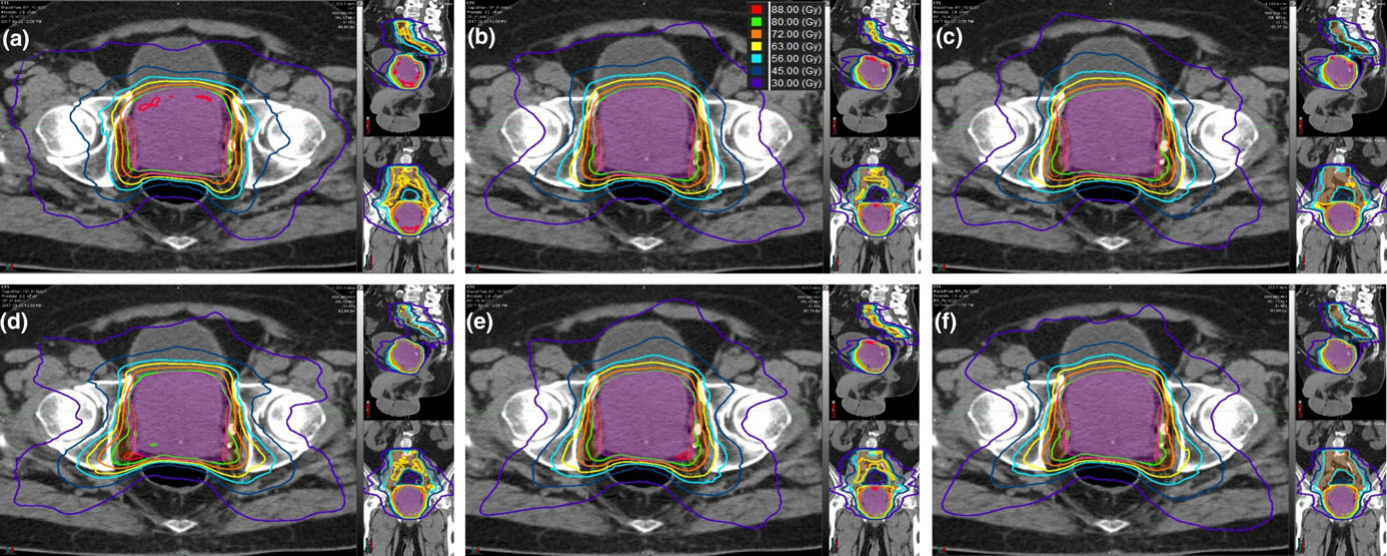
Example patient plans for prostate plus lymph nodes utilizing (a) Expert planner, (b) the KBP Model with only prostate plus lymph node cases (PLB), (c) full prostate and prostate with lymph nodes KBP Model (PPLN), and the three reduced KBP libraries 31 total mixed prostate cases and prostate plus lymph node cases (d) P11PLN20, (e) P16PLN15, and (f) P20PLN11.

**Figure 3:**
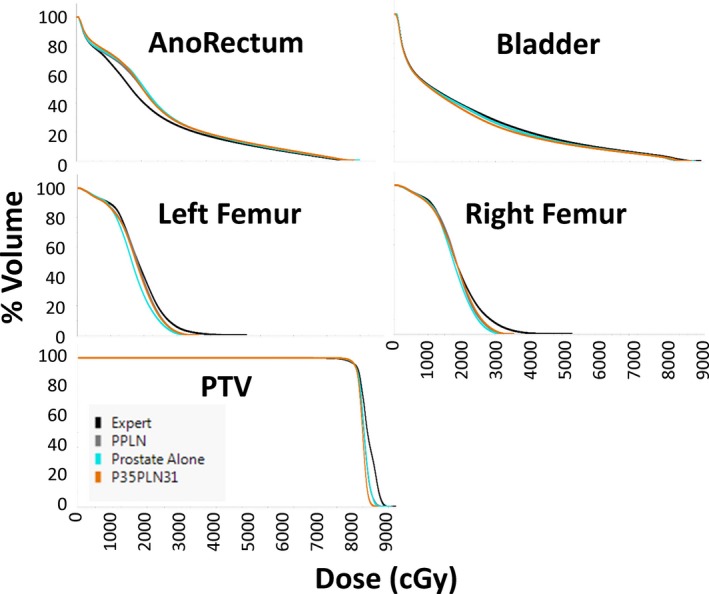
DVHs averaged over all ten plans of a type for the expert, the Prostate plus Pelvic Lymph Node full KBP library (PPLN), the Prostate alone KBP library (Pr Alone) and the KBP library with 35 prostate cases and 31 prostate plus lymph nodes cases (P35PLN31).

**Figure 4:**
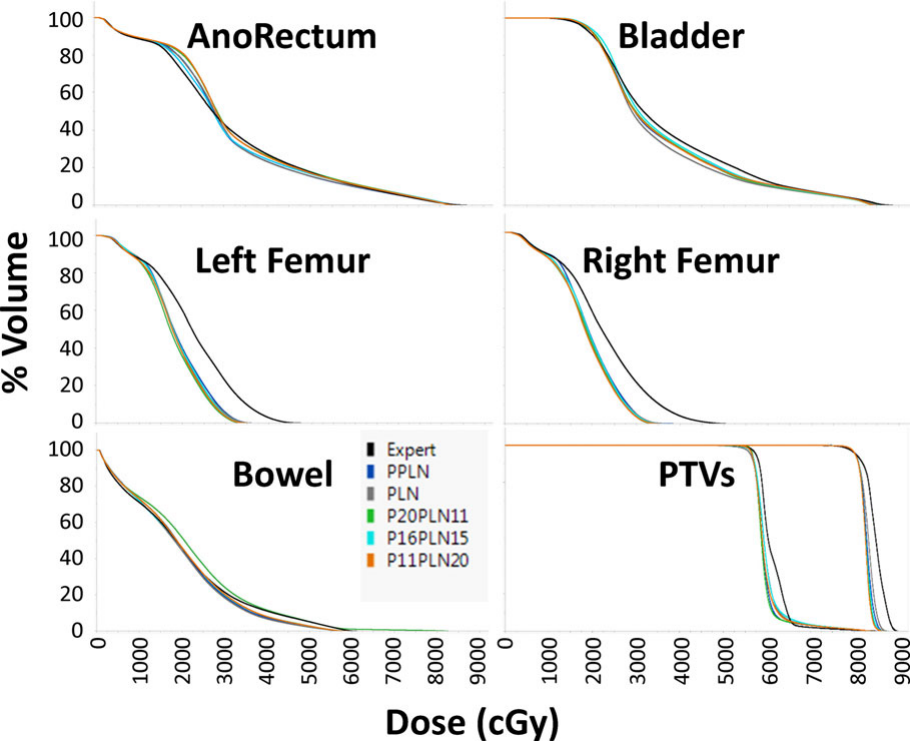
DVHs averaged over all ten plans of a type for the expert, the Prostate plus Pelvic Lymph Node full KBP library (PPLN), the Prostate alone KBP library (Pr Alone) and the three reduced KBP libraries with 31 total mixed prostate cases and prostate plus lymph nodes cases (P11PLN20, P16PLN15 and P20PLN11).

Figure 1 is an example plan for the treatment of prostate alone. The expert plan exhibits slightly better dose distributions around the anorectum, but the KBP models demonstrate much better sparing of the femoral heads. Otherwise, there are only few differences between the plans. Figure 2 is an example plan for the treatment of prostate plus lymph nodes. For these plans, each of the KBP models has better sparing of the femoral heads with very few other differences between plans for each model type. Figures 3 and 4 are the mean dose volume histograms (DVHs) over all 10 plans of a planning type (i.e., expert or KBP model) for each OAR and PTV for prostate alone (Fig. 3) and prostate plus lymph nodes (Fig. 4) plans.

Figure 3 echoes what is seen in the example isodose distribution: the expert planner has slightly better dose distributions through the intermediate dose range (around 750–4000 cGy) than the KBP plans for the anorectum while the maximum doses to the femoral heads is higher for the expert plans. Bladder doses are more or less identical for all prostate alone plans. For the PTVs, the KBP plans, overall, have a steeper dose fall‐off than the expert plans.

Figure 4 shows that the femoral heads are better spared with the all the various KBP models than the expert plans. It also shows that the dose fall‐off for both the PTVs was less steep for the expert plans than it was for any of the KBP plans. Otherwise, the sparing of OARs were very similar, though it could be said that the expert anorectum volumes at doses in the range of 1500–3000 cGy were lower (and therefore, superior), but were superseded around an inflection point at 3000 cGy such that the KBP plans had lower anorectum volumes up to around 6500 cGy when the volumes more or less even out.

## 4 DISCUSSION

This study demonstrated that a KBP model with mixed plan types (Pr or PLN vs PPLN) does as well as a single purpose model. KBP library models are a good way to incorporate the expert knowledge base found from prior patient planning and rapidly utilize this knowledge to predict planning outcomes for new patients. Many groups have utilized in‐house created models applied to a variety of body sites.^1^, ^3^, ^7^, ^8^, ^15^, ^16^, ^17^, ^18^, ^19^, ^20^, ^21^, ^22^, ^23^ Most have started with prostate planning due to the simplicity of the site in terms of ease of planning and the bonus of having a database that includes a large number of consistent plans from which to draw. Our use of plans from various in‐house clinical trials allowed us to have a body of patients with very consistent patient contouring, a variety of patient sizes/shapes, and very well thought out, stable plans.

Some work that was done by the group prior to this work was to refine models (a), (b) and (c) by copying the models, removing any dosimetric outliers from the data set, replanning the patient with the model, then reincorporating the new plan into the model. The difference in plans done with these refined models and the original ones were insignificant, so we decided to stay with the original models. This finding is in line with Delaney, et al.^24^ and Hussein, et al.^3^ who showed that the removal of outliers from a good quality model training set did not have a significant impact on the final plan quality.

The work here shows that a KBP model with mixed plan types (prostate alone vs prostate plus pelvic lymph nodes) does as well as a single purpose model. This indicates that a robust KBP model can be created from a variety of plan types for a particular region of the body. Admittedly, prostate is a fairly simple model to start with (definitely the reason there are so many papers on getting started with a prostate KBP model), and this model likely scales fairly well to a problem like larynx alone vs larynx plus nodal regions. The broader question is whether it scales to more complex questions of head and neck where there are multiple dose levels incorporated into the planning, or one‐sided vs bilateral treatment types. Obviously multiple plans of a particular type would be needed to build a model capable of creating a reasonable treatment plan, but would a model that is more focused do better in this type of case? The data here indicates that the more focused model would not necessarily do better or worse, though the details of this question is left to future work. The information seen here shows that the broader model will do as well, and the number of plans mimicking a particular geometry would not need to be as high in order to create a robust KBP library capable of planning on a large body of cases.

Interestingly, the size of the models (66 vs 97 plan models, and 33 vs 97 plan models) made no significant difference in the plan quality for either the Pr or the PLN cases. This could be a case of quality in — quality out. That is to say, the quality of the cases that made up the database were consistent and good enough as to render having 2 or 3 times the number of plans unnecessary. As well, for PLN cases the ratio of plans in the model made no difference in the model's ability to create a viable plan. Again, this possibly speaks to the quality of the plans in the model that a random sampling gave us good models in all cases.

## 5 CONCLUSIONS

This study indicates that a combined KBP library model library performs as well as a single purpose model, especially for the more complex plans. This indicates that a good prostate cancer model can be created with a mix of plans for treating prostate alone and prostate plus pelvic lymph nodes, and this model will perform well, even for more complex treatment geometries. The general feeling is that this result could be extended to other body sites and plan types, though further investigation is warranted.

## CONFLICTS OF INTEREST

Dr. Elizabeth Bossart received travel support to attend the Varian Research Partners meeting from Varian Medical Systems.
